# RNA allows identifying the consumption of carrion prey

**DOI:** 10.1111/1755-0998.13659

**Published:** 2022-06-20

**Authors:** Veronika Neidel, Daniela Sint, Corinna Wallinger, Michael Traugott

**Affiliations:** ^1^ Applied Animal Ecology, Department of Zoology University of Innsbruck Innsbruck Austria

**Keywords:** food webs, molecular diet analysis, scavenging, top‐down control, trophic cascades, trophic linking

## Abstract

Facultative scavenging by predatory carnivores is a prevalent but frequently underestimated feeding strategy. DNA‐based methods for diet analysis, however, do not allow to distinguish between scavenging and predation, thus, the significance of scavenging on population dynamics and resource partitioning is widely unknown. Here, we present a methodological innovation to differentiate between scavenging and fresh prey consumption using prey RNA as a target molecule. We hypothesized that the rapid post‐mortem breakdown of RNA in prey tissue should lead to a significantly lower detection probability of prey RNA than DNA when carrion rather than fresh prey is consumed. To test this hypothesis, ground beetles (*Pseudoophonus rufipes* [De Geer]) were offered either fresh or 1‐day‐old dead *Drosophila melanogaster* fruit flies (carrion). The detectability of prey RNA and DNA in the beetles' regurgitates was assessed with diagnostic *Drosophila‐*specific RT‐PCR and PCR assays at 0, 6, 12, 24 and 48 h post‐feeding. After fresh fly consumption, prey RNA and DNA were detectable equally well at all times. When carrion prey was consumed, the detection strength of prey RNA immediately after feeding was significantly lower than that of prey DNA and reached zero in most samples within 6 h of digestion. Our findings provide evidence that prey RNA allows distinguishing between the consumption of fresh and scavenged prey, thereby overcoming a long‐known weakness of molecular diet analysis. The assessment of prey RNA offers a generally applicable approach for examining the importance of scavenging in food webs to unravel its functional consequences for populations, communities, and ecosystems.

## 1 INTRODUCTION

Carrion prey is a food source providing nutrients at a comparatively low demand in energy input and, therefore, is highly sought‐after by many animals (Barton et al., 2019). Besides obligate scavengers, also a wider range of predatory vertebrates (DeVault et al., 2003; Pereira et al., 2014) and invertebrates feed on animal carcasses (Barton et al., 2019; Foltan et al., 2005; Moleon & Sanchez‐Zapata, 2015; Wilson et al., 2010). Thus, scavenging plays an important role in the dynamics and functioning of ecosystems (Moleon & Sanchez‐Zapata, 2015). As an additional food source, carrion offers predators easily accessible energy that can result in increased top‐down control (Polis & Strong, 1996; Wilson & Wolkovich, 2011). Facultative scavenging interconnects the feeding guilds of scavengers and predators, which in theory are often strictly separated (Mattisson et al., 2016; Moleon & Sanchez‐Zapata, 2015), and, as a form of multichannel‐feeding (Wolkovich et al., 2014) or omnivory, blurs the lines between different trophic levels (Polis & Strong, 1996). Moreover, feeding interactions with detritus, the collective biomass of dead organic material including carrion, are considered to stabilize food webs (Moore et al., 2004; Polis & Strong, 1996).

While data on the nutritional value of carrion is scarce, it is likely to be variable because its acceptance as food for predators is not indefinite, and decreases with increasing levels of decay (Foltan et al., 2005; Juen & Traugott, 2005). Mellard et al. (2021) argue that it makes sense for a predator to approach carrion even if its energy content is unknown or low, because the profitability, which is a function of energy content, handling time, search time and prey mobility, will in many cases be better for carrion than for live prey. Experimental studies of invertebrates (Foltan et al., 2005; Mair & Port, 2001) and empirical data of vertebrate predators (Mattisson et al., 2016; Pereira et al., 2014) support this assumption, as predators have been found to prefer carcass over live prey in cases where prey defence was highly efficient but predation and scavenging were equally likely if vulnerable prey individuals were available. Thus, changing availability of vulnerable prey could even lead to seasonal variability of carrion prey use within the same predator species (Pereira et al., 2014).

Many trophic studies, however, do not account for the possibility that ingested animal prey could be the result of scavenging rather than active predation (Wilson & Wolkovich, 2011). Such incorrect linkage in food webs can lead to an overestimation of top‐down control by predators because the consumption of dead individuals does not directly affect prey populations (Foltan et al., 2005; González‐Chang et al., 2016; Sheppard & Harwood, 2005; Sunderland, 1996). Scavenging is, however, often difficult to monitor, partly because carrion prey has a short retention time in the field due to its quick consumption and decay (Fellers & Fellers, 1982; Seastedt et al., 1981; Sugiura et al., 2013).

Depending on the research question and ecological context, different methods for the study of trophic interactions under field conditions are available apart from direct feeding observations such as stable isotope and fatty acid analysis (Boecklen et al., 2011; Ruess & Chamberlain, 2010), or the identification of prey remains in field‐collected gut content, faeces, or regurgitates (Symondson, 2002; Traugott et al., 2013). Prey remains that are beyond the scope of visual determination can be identified with molecular methods (Traugott et al., 2021). Monoclonal antibodies have been used to study foraging strategies by providing carrion and live prey with different protein labels that made them distinguishable (Mansfield & Hagler, 2016; Zilnik & Hagler, 2013). However, the prey's own antigens do not allow to differentiate between scavenging and predation (Calder et al., 2005). This restricts the approach to experimental setups because it requires previously manipulated prey items. Also, when applying DNA‐based approaches DNA of fresh prey and carrion is detected equally well in the gut content of carabid beetles (Foltan et al., 2005; Juen & Traugott, 2005). This indicates that DNA analyses alone are not sufficient to make a statement about the prey capture strategy.

Unlike DNA, RNA breaks down quickly after death (Sidova et al., 2015). In forensic sciences, RNA has therefore been used as a target molecule for determining post‐mortem intervals since the 1980s (Bauer, 2007). Recently, a growing number of studies have explored the benefits of RNA complementing DNA‐based studies for biomonitoring (Cristescu, 2019). For example, the shorter detection intervals of environmental RNA (eRNA) in comparison to environmental DNA (eDNA) in water samples, allow for the differentiation between current and former marine communities (Wood et al., 2020).

Both, the limited time interval of detection within tissues and in the environment (Wood et al., 2020; Yasojima et al., 2001), make RNA a promising target for distinguishing between the consumption of live and carrion prey. Using RNA as a target molecule in trophic studies has been explored only in theory, but to the best of our knowledge, not in practice (Nielsen et al., 2018).

Here we test, for the first time, the practicability of targeting prey RNA for differentiating between fresh and scavenged prey. Based on the expected faster deterioration of RNA compared to DNA, we hypothesize that the prey type – fresh prey or carrion ‐ (1) will not make a difference for prey DNA detection probability and (2) relative prey DNA content in diet samples, but that (3) prey RNA detection probability as well as (4) relative prey RNA content will be lower in diet samples in the case of carrion prey consumption.

We conducted feeding experiments with carabid beetles, a group of insects considered important for biocontrol in agricultural fields, and previously studied by DNA‐based diet analysis regarding their scavenging behaviour (Foltan et al., 2005; Juen & Traugott, 2005). The carabid *Pseudoophonus (Harpalus) rufipes* (DeGreer, 1774) is an omnivorous species, that can occur in high numbers in arable land (Luff, 1980; Sunderland, 2002). While the larvae of this beetle are granivorous, the adults also feed on a range of invertebrate taxa, for example, aphids and dipterans (Loughridge & Luff, 1983; Sunderland, 1975) and engage in both, active predation and facultative scavenging (von Berg et al., 2012). Fruit flies were used as the experimental prey in the present experiments, because adult dipterans are considered a frequently taken prey and high‐quality food for generalist carabid beetles native to agroecosystems (Sunderland, 1975, 2002; Toft & Bilde, 2002).

In feeding experiments, the beetles were offered either fresh or carrion prey, and their gut content was screened at different points in time after feeding to reveal the detectability of prey DNA and prey RNA. Our results indicate that an analysis of both molecules, DNA and RNA, in parallel screenings of dietary samples, allows not only for a specific prey identification but also for a distinction between the prey types. As such, our findings provide a significant methodological advancement with broad applicability in trophic studies of facultative scavengers.

## 2 MATERIALS AND METHODS

### 2.1 Experimental consumers: adult *Pseudoophonus* rufipes carabid beetles

Adult beetles of the species *Pseudoophonus rufipes* were collected in July 2019 by pitfall trapping in Aldrans, near Innsbruck, Austria. For the duration of our study, carabids were kept individually in plastic cups with screw‐top lids (56 mm diameter × 71 mm height), containing moistened tissue paper. Cups were stored in a climate cabinet with an artificial day‐night rhythm of L:D 14:10 h and an alternating temperature regime of 22 and 12°C, respectively. They were ventilated daily and moistened tissue was renewed every second day. The beetles were maintained on a diet of mealworms (*Tenebrio molitor*, L.) until being starved for 4 days before the feeding experiments. At the end of the study, all carabids were released unharmed to a suitable habitat.

### 2.2 Experimental prey: fruit flies *Drosophila melanogaster*

Common fruit flies, *D. melanogaster* (Meigen, 1830) (variation “curly‐winged”, strain SCO/CyO) served as animal prey. Fly colonies were obtained from the laboratory rearing of the Molecular Biology Division, Medical University Innsbruck, Austria, and propagated on a cornmeal‐yeast diet in the same climate cabinet described above until enough flies had emerged.

Shortly before the feeding experiments, adult flies were freeze‐killed at −16°C. To simulate two prey types in the experiment, frozen flies were offered to carabids either immediately after defrosting (subsequently referred to as fresh prey) or after decaying in the climate cabinet for 24 h (subsequently referred to as carrion prey).

### 2.3 Feeding experiments: consumers feed on fresh or carrion prey

For the feeding experiments, carabids were placed individually in clean screw‐lid cups, containing one fly, representing either fresh or carrion prey, and a drop of water. Carabids were allowed to feed for 1 h in darkness in the climate cabinet. In case carabids had not consumed the fly, they were granted another hour of feeding, up to three times. If carabids had consumed the entire fly, they were included in the experiment and stimulated to regurgitate at different points in time after feeding. To do so, beetles were placed individually headfirst in 1.5 ml microtubes. Regurgitation was then elicited through heat stress by repeatedly dipping the tip of the tube into hot water for less than a second (Wallinger et al., 2015). The maximum feeding time of 3 h was needed by only three beetles, all of which were assigned to the fresh prey treatment with regurgitation after 6 h. Batches of 10–14 regurgitates were collected per prey type (fresh or carrion prey) at 0, 6, 12, 24, and 48 h after feeding (Table S1). Regurgitates were immediately placed in a freezing rack for the time required to collect regurgitates from all beetles in the respective cohort and then transferred to storage at −80°C until further processing.

### 2.4 DNA & RNA extraction

Regurgitates were mixed with 200 μl DNA/RNA Shield (Zymo Research) and total nucleic acids were extracted using the IndiSpin Pathogen Kit (Indical Bioscience GmbH) on a BioSprint96 automatic extraction platform (Qiagen) according to the manufacturer's instructions (Indical Biosciences, https://www.generon‐food‐safety.com/product/indispin‐pathogen‐kit/) with minor alterations: the VXL‐buffer mastermix was prepared without carrier RNA and total nucleic acids were eluted in molecular grade water instead of AVE buffer to facilitate subsequent DNA digestion.

While the presence of RNA does not interfere with DNA detection, co‐present DNA will usually confound the analysis of RNA with reverse transcription (RT)‐PCR. Thus, to allow for a comparison of the presence of prey DNA and RNA in the samples, the extracts of total nucleic acids were split, and one portion was treated with DNase to get pure RNA extracts as described below. Total DNA/RNA extracts will further be referred to as “DNA extracts” and digested split samples as “RNA extracts”. DNA/RNA were extracted in two independent rounds. The first batch included 92 samples; the second batch had 23 samples. In each batch, two extraction negatives (molecular grade water) were included to check for possible cross‐contamination.

### 2.5 Digestion of DNA in RNA samples

The Monarch RNA Cleanup Kit (New England BioLabs Inc.) was used as follows: a mix of 22.2 μl DNA/RNA extract, 2.5 μl DNase I reaction buffer and 0.3 μl DNase I enzyme was prepared on ice before incubation at 37°C for 20 min. Then, 0.5 μl of 0.25 M EDTA was added to each sample, followed by heat inactivation of DNase at 75°C for 15 min. Incubation and heat inactivation were both done in an Eppendorf Mastercycler nexus (Eppendorf AG).

The digested samples were thereafter screened for residuals of *Drosophila* DNA target molecules with the PCR protocol described for prey DNA detection below, but with a stricter detection threshold: in case any *Drosophila* DNA was still amplified in an RNA sample, the DNA digestion was repeated with more enzyme and a longer incubation. To do so, 22.0 μl fresh DNA/RNA extract, 2.5 μl DNase I reaction buffer, 0.5 μl DNase I enzyme were incubated at 37°C for 30 min. Inactivation and screening for residual DNA were the same as above. After this extended DNA digestion, no *Drosophila* DNA was detectable in any of the RNA samples.

### 2.6 Molecular analysis: capillary electrophoresis PCR (celPCR) assay for detection of prey DNA and reverse transcription (RT)‐celPCR assay for detection of prey RNA

DNA extracts were screened with the genus‐specific primer pair Droso‐S391 (5′‐AAATAACAATACAGGACTCATATCC‐3′) and Droso‐A381 (5′‐GTAATACGCTTACATACATAAAGGTATA‐3′), targeting a 240 bp fragment of the nuclear 18S rDNA of *Drosophila spp*., that can detect initial prey DNA amounts of as little as 0.02 pg (Wolf et al., 2018). PCR was done with a total volume of 10 μl, containing 1× Multiplex PCR Master Mix (Qiagen), 0.5 μM of each primer, 5 μg BSA, 2 μl DNA extract and molecular grade water. The thermocycling protocol was 15 min at 95°C, 35 cycles of 30 s at 94°C, 90 s at 62°C and 60 s at 72°C, followed by a final elongation at 72°C for 10 min (Wolf et al., 2018).

The RNA extracts without residual *Drosophila* DNA were screened with RT‐PCRs, also employing the primer pair Droso‐S391/Droso‐A381. For RT‐PCR, 10 μl reactions contained 2 μl RNA extract, 1× OneStep RT‐PCR Buffer (Qiagen), 400 μM of each dNTP, 0.6 μM of each primer, 0.4 μl Qiagen OneStep RT‐PCR Enzyme Mix and molecular grade water. Thermocycling included a reverse transcription step of 30 min at 50°C followed by denaturation at 95°C for 15 min, 35 cycles of 94°C for 30 s, 62°C for 60 s and 72°C for 60 s, and final elongation at 72°C for 10 min.

All samples were screened without technical replicates. Within each PCR/RT‐PCR, one negative control (molecular grade water) and one positive control (fly DNA/RNA) were included to check for carry‐over contamination and amplification success, respectively.

Undiluted PCR/RT‐PCR products were visualized by capillary electrophoresis on the QIAxcel Advanced System (Qiagen), with the method AM320 and an injection time of 30 s. Peaks above a threshold of 0.1 relative fluorescent units (RFU) were considered positive, and the RFU value of each PCR product was recorded as a relative estimate of DNA or RNA content in the sample. In the extraction batch 2, which was comprised of regurgitates taken after 24 h (8 of 13 samples), and all taken after 48 h (*n* = 14) of beetles fed with carrion prey, one of the extraction negatives tested positive for *Drosophila* DNA and RNA. To allow for all samples to be used in the analysis despite this contamination, we subtracted the RFU value measured in the extraction‐negative from the values of the samples in batch 2 (total *n* = 23) before any further data processing. All PCR negative controls, however, were clean.

### 2.7 Statistical analysis

All analyses were performed in R version 3.5.0 (R Core Team, 2018). First, all prey RNA and DNA signals below the threshold of 0.1 RFU were set to zero. The resulting signal strength data set, reflecting our relative estimate of prey RNA and DNA fragments, had a non‐normal distribution and unequal variances, which was revealed by the Shapiro–Wilk Normality (SWN) test and the Levene test, included in the r‐package “car” (Fox & Weisberg, 2019). Therefore, nonparametric statistical tests were used for the direct comparison of mean RFU values. Signal strengths of prey RNA or prey DNA were compared between prey types for each point in time after feeding with Wilcoxon rank‐sum (WRS) tests. Within the same prey type, RNA and DNA signals were compared at each sampling point with the Wilcoxon signed rank (WSR) test for matched pairs. Previous to this test, samples with a difference of zero between DNA‐ and RNA‐signal strength were excluded. Effect sizes “*r*” for WRS and WSR tests were calculated as $\;r=\frac{Z}{\sqrt{N}}$ (Field et al., 2012), where “*Z*” is the *z*‐score of the statistical test and “*N*” is the number of observations in the comparison.

Detection probabilities of *D. melanogaster* DNA and RNA in the regurgitates of *P. rufipes* were analysed by generalized linear models (GLM) with a LOGIT link function. DNA and RNA detections were therefore translated into a binary response variable (cutoff ≥0.1 RFU) and digestion time was used as a predictor for detection probability of each target molecule for each prey type, separately. To test for the effect of prey type, the overall detection probabilities of prey DNA and prey RNA were calculated in combined models using the variables digestion time and prey type as predictors. Using the function “lrtest” of the r‐package “lmtest” (Zeileis & Hothorn, 2002), likelihood ratio tests were applied to compare models containing either one predictor, two predictors, or their interaction. Both, Hosmer‐Lemeshow Goodness of Fit (HL‐GOF) and Akaike information criterion (AIC) were considered for model selection.

We calculated a within‐sample ratio of RFU_RNA_:RFU_DNA_ for samples with a DNA detection signal strength greater zero. SWN‐tests indicated that the ratio data was also not normally distributed. The pairwise comparisons of the treatments at different times after feeding were, thus, conducted with the nonparametric Wilcoxon rank sum (WRS) test.

## 3 RESULTS

A total of 116 regurgitates were screened and used for data analysis (Table S1). Prey RNA and DNA were amplified above the threshold of 0.1 RFU in 49 and in 77 of the regurgitate samples, respectively.

### 3.1 Prey RNA was detected more often and with consistently higher signal strengths in regurgitates after fresh compared to carrion prey consumption

**FIGURE 1:**
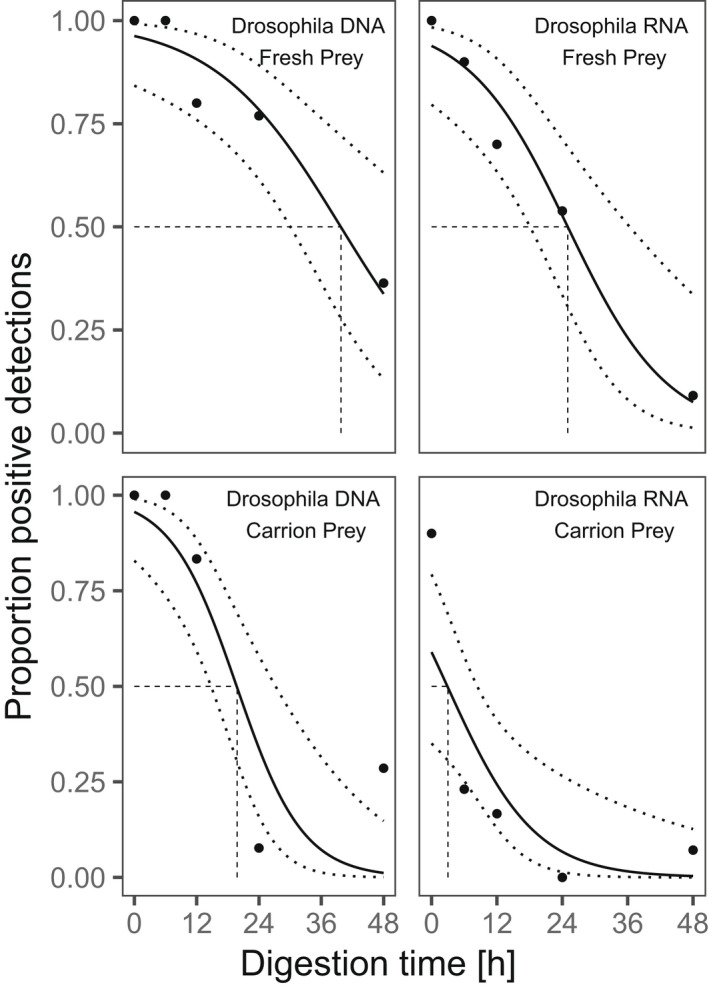
Detection rate and probability of *Drosophila melanogaster* DNA (left panels) and RNA (right panels) in regurgitates of *Pseudoophonus rufipes*, 0, 6, 12, 24, and 48 h after feeding on one fresh (top) or one 24‐h‐dead fly (bottom). The proportion of samples positive for the molecular target (●) is plotted along the detection probability (solid lines) with 95% CIs (dotted lines) calculated with generalized linear models (GLM)

**FIGURE 2:**
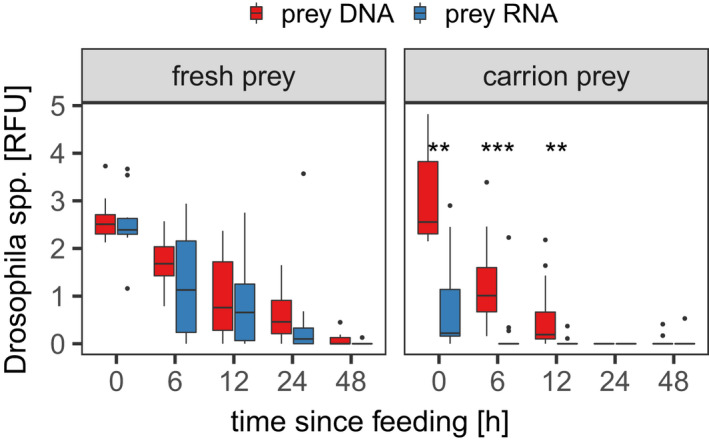
Comparison of prey DNA and prey RNA signal strengths, measured as relative fluorescent units (RFU), in regurgitates of *Pseudoophonus rufipes* within the same prey treatment, fresh (left panel) or carrion prey (right panel) after 0, 6, 12, 24 and 48 h of digestion. Significant differences revealed by Wilcoxon signed‐rank test are indicated with (***) for *p* < .001 and (**) for *p* < .01

Prey RNA was detected in 63% of all regurgitates within the fresh prey treatment but only in 24% in the carrion prey treatment. At each post‐feeding time‐point, the proportion of samples with positive prey RNA detection was higher when fresh prey rather than carrion prey had been consumed (Figure 1, Table S2). Signal strengths of *Drosophila*‐specific prey RNA ranged from 0 to 3.67 RFU. The relative amount of prey RNA, measured as signal strength (RFU values), was frequently higher in regurgitates with fresh than in those with carrion prey (Figure 2, Table 1).

**TABLE 1:** Pairwise comparison of different prey types: Relative amount (signal strengths; relative fluorescent units [RFU]) of *drosophila*‐specific (a) prey RNA and (b) prey DNA within regurgitates of *Pseudoophonus rufipes*, including pairwise comparisons of different prey types at different time points after feeding via Wilcoxon rank‐sum tests, with the test statistic *W*, the effect size *r*, the significance value *p* (*<.05, **<.01, ***<.001) and sample number of the comparison *n*

	Digestion time	Fresh prey	Carrion prey	*W*	*p*	*r*	*n*
		RFU mean ± sd	RFU mean ± sd				
a. RNA	0	2.52 ± 0.7	0.84 ± 1.05	85	.008**	−0.59	20
	6	1.26 ± 1.11	0.22 ± 0.62	111	.003**	−0.63	23
	12	0.87 ± 0.94	0.04 ± 0.11	98	.005**	−0.60	22
	24	0.43 ± 0.97	0 ± 0	NA	NA	NA	26
	48	0.01 ± 0.04	0.04 ± 0.14	NA	NA	NA	25
b. DNA	0	2.61 ± 0.48	3.12 ± 1.06	41	.496	−0.15	20
	6	1.74 ± 0.58	1.19 ± 0.93	97.5	.044*	−0.42	23
	12	1 ± 0.91	0.56 ± 0.75	80	.186	−0.28	22
	24	0.6 ± 0.57	0 ± 0	NA	NA	NA	26
	48	0.08 ± 0.14	0.04 ± 0.12	NA	NA	NA	25

*Note:* Test statistics omitted for groups with corrected values (24, 48 h).

### 3.2 Prey DNA detection probabilities are similar between prey types, signal strengths are lower for carrion after 6 h post‐feeding and longer

Prey DNA was detectable in 77% of all regurgitates with fresh prey, and in 56.5% of regurgitates with carrion prey. The proportion of samples positive for prey DNA was equally high at 0, 6, and 12 h after feeding, but lower after more than 24 h (Figure 1, Table S2). The signal strength of *Drosophila*‐specific PCR amplicons ranged from 0 to 4.82 RFU for prey DNA. Except for 0 h, we frequently detected lower signal strengths of prey DNA after carrion prey consumption (Table 1).

### 3.3 Greater difference between RNA and DNA signal strength in samples of carrion prey consumption

RFU values of *Drosophila* prey DNA and prey RNA in regurgitates were similar to each other at the same time after feeding when fresh prey was consumed. After carrion prey consumption, however, RNA signals were significantly weaker than DNA signals (Figure 2 & Table S3). While detections decreased throughout digestion, no significant differences were found at more than 12 h post‐feeding between prey DNA and prey RNA due to the overall low detection rates.

### 3.4 Negative effect of increasing digestion intervals on detection probabilities and signal strengths

For both prey types, RNA and DNA detection probabilities significantly decreased with digestion time (Table S4). Individual models for *Drosophila* RNA and DNA, respectively, predicted a drop of detection probabilities below 50% after digestion times of 24.9 and 39.7 h for fresh prey, and after 2.9 and 19.7 h for carrion prey (Figure 1).

Overall, the detection probability of prey RNA was significantly negatively affected by both, digestion time and prey type (Table S4). Within the same prey type, the odds of RNA detection decreased by 11% with each passing hour (CI 95%: 0.16, 0.07, *p* < .001). At a fixed time after feeding, the odds of detecting prey RNA in regurgitates with carrion was 92% lower than in samples with fresh prey (CI 95%: 0.98, 0.77, *p* < .001).

For DNA, the overall detection probability significantly decreased over time of digestion and was lower after carrion prey consumption. The odds ratio of DNA detection was 83% (CI 95%: 0.95, 0.46, *p* = .005) lower after carrion prey consumption. The odds of DNA detection decreased by 10% (CI 95%: 0.14, 0.07, *p* < .001) per passing hour of digestion.

### 3.5 RFU_RNA_ :RFU_DNA_ ratios

**FIGURE 3:**
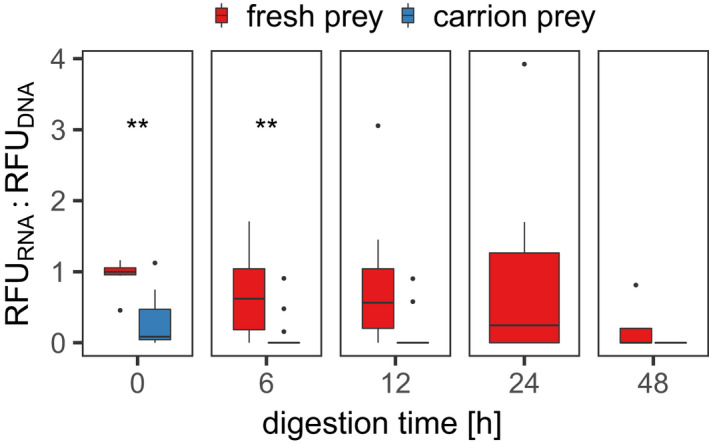
Boxplots of the ratios RFU_RNA_:RFU_DNA_, measured for capillary electrophoresis PCR (celPCR) products of regurgitates of *Pseudoophonus rufipes* 0, 6, 12, 24 and 48 h after consumption of one fresh (red) or one carrion fruit fly (blue). Significant differences between ratios of different prey types as revealed by Wilcoxon rank sum test are indicated with (**) for *p* < .01. All samples with carrion prey were negative for the target prey at 24 h after feeding

The in‐sample ratios of the signal strengths produced for prey RNA and DNA were higher in fresh prey than in carrion prey, but mean values differed between time points after feeding (Figure 3). The differences between the ratios of fresh and carrion prey were statistically significant at 0 (WRS, *W* = 89, *r* = −.66, *p* = .003) and 6 h after feeding (*W* = 109, *r* = −.58, *p* = .006) (Table S5). At 24 h, none of the carrion prey samples tested positive for the target prey, thus no ratios could be calculated for this group.

## 4 DISCUSSION

We demonstrated that the detection probability and signal strength of prey‐specific RNA was significantly lower when carrion prey had been consumed. This supports our core hypotheses that prey RNA detection success is reduced in carrion prey due to a continuous breakdown of RNA within the carcass. Surprisingly, detection of prey RNA and DNA worked equally well when fresh prey had been consumed, conflicting with the common expectation that RNA should deteriorate faster than DNA (Cristescu, 2019). We chose the prey type “fresh prey” – recently freeze‐killed flies – as a compromise between providing living prey (predation) and requiring the prey item to be consumed quickly. We assume that RNA detectability for this prey type is similar to the one for predation because the RNA content of a fly will only decrease after its death. If consumption of the freshly killed fly can be distinguished from consumption of carrion prey based on its RNA content, the difference should be even more pronounced in field situations when living prey is consumed. Thus, a direct comparison between the relative amount of prey RNA and DNA within a sample can indicate whether a feeding interaction is the result of scavenging or predation. As such, our work provides a first proof of concept for a new method to assess the prevalence of scavenging in the field.

The lack of a suitable method for identifying scavenging as such in diet analysis has long been considered a critical gap of knowledge (Juen & Traugott, 2005; Lovei & Sunderland, 1996; Nielsen et al., 2018), and a problem for assessing the functional and community‐wide implications in predator–prey interactions involving vertebrate (Egeter et al., 2019) and invertebrate consumers in terrestrial (Foltan et al., 2005; Juen & Traugott, 2005) and aquatic ecosystems (Beasley et al., 2012). Analysing prey RNA will help to unravel carrion feeding in real‐world food webs, without a need of quantifying the actual availability of carrion (Barton et al., 2019). Importantly, it will allow quantifying the frequency of facultative scavenging and help to re‐evaluate its role for ecosystem functioning (Wilson & Wolkovich, 2011). Moreover, gut content screenings aimed at the consumption of live prey to estimate top‐down control will benefit from targeting prey RNA instead of DNA because of its lower chance to record carrion prey links. We anticipate that the method should be evaluated for other dietary sample types, such as whole‐body samples or faeces.

To bring prey RNA methods to the field, several methodological considerations can be derived from the existing literature on best practice for diet analysis of prey DNA. For example, appropriate sample handling to avoid DNA cross‐contamination or standard procedures for protocol optimization and the inclusion of technical controls (King et al., 2008; Traugott et al., 2021). Sample preservation is a crucial step for obtaining meaningful results from RNA (Laroche et al., 2017), which needs to be considered when using the prey RNA approach. For regurgitates, the immediate freezing of samples after collection and storage until further processing at −80°C for 12 months was a suitable method. Alternatively, the use of buffer solutions for sample preservation allows postponing sample freezing, which might be of advantage during field collections. Faecal samples for DNA‐based diet analysis, for example, have been preserved successfully in DNA/RNA Shield (Zymo Research) (Loo et al., 2019) and RNAlater Stabilization Solution (Invitrogen) (Kartzinel & Pringle, 2015; Vo & Jedlicka, 2014). DNA/RNA Shield allows for samples to be stored at temperatures below 25°C for up to 30 days, and infinitely at −20°C, and samples preserved in RNAlater can be stored at 4°C for up to a month. However, there are certain limitations to this way of sample preservation. RNAlater, for example, needs to be removed from samples before RNA extraction for most protocols. Thus, for regurgitate samples DNA/RNA Shield might be a better option. Further, according to the manufacturer's protocol of RNAlater, tissue samples need to be smaller than 0.5 cm and proper sample penetration is only achieved after overnight incubation at 4°C. Therefore, it is probably not suited for whole‐body samples of arthropods. Even for arthropod tissue, RNA quality is better in samples flash‐frozen with liquid nitrogen than in samples preserved in RNAlater (Kono et al., 2016). Therefore, we assume that for whole‐body samples the immediate transfer to a freezer after sample collection might be the best option to prevent the breakdown of RNA in consumers' guts.

The proposed method of combined RNA and DNA analysis is more expensive and laborious than prey DNA or prey RNA analysis alone. This is, first, because two separate subextracts need to be produced for the within‐sample comparison of prey RNA and DNA. This can be achieved by a coextraction of both nucleic acids, followed by an additional step of DNA digestion in a split fraction, which is an approach also frequently found in eRNA studies (Marshall et al., 2021; Wood et al., 2020). Second, RNA needs to be transcribed to cDNA for PCR. Depending on the chosen protocol and kit, the cDNA can be synthesized in advance, as shown by Adamo et al. (2021) and Laroche et al. (2018), or in a single‐tube protocol as a part of the PCR, as described here. Finally, to compare the presence and amount of prey RNA and DNA, each sample needs to go through at least two PCRs, and additionally, RNA extracts need to be screened for residual DNA, as the same primers should be used for RNA and DNA detection to facilitate comparability. Unfortunately, the use of an exon‐exon junction primer for the amplification of RNA is no alternative for a within sample comparison of RNA and DNA, as employing different assays will introduce additional variability to the analysis, which can best be avoided by using the same assay to detect RNA and DNA. It is difficult to estimate the exact additional work time required, as this depends on the available laboratory infrastructure. However, performing a simple enzymatic digestion and two additional PCRs per sample are a manageable effort. The cost of consumables is about twice of that of comparable DNA analyses and can be estimated at ~5 € per sample for testing for both prey DNA and prey RNA (excluding costs for labour). A streamlining of all processes might, however, reduce the time and costs considerably.

For diet analysis, primers on various marker gene regions have been published (King et al., 2008). As described above, cDNA is synthesized in vitro from RNA before PCR. It is therefore likely that PCR primers developed for DNA‐based diet analysis can be applied for prey RNA assays without modification if the targeted gene regions are transcribed to RNA within the cells. This is the case in functional regions, such as the protein‐coding cytochrome c oxidase subunit I (COI) in the mitochondrial genome, or the ribosomal RNA gene (18 s rDNA), which we used here. Both marker regions have successfully been targeted in eRNA/eDNA studies (Marshall et al., 2021; Wood et al., 2020). Also the noncoding internal transcribed spacer region 2 (ITS2) is transcribed to RNA within the cell (Coleman, 2009), and was already used as a molecular marker in an eRNA study (Adamo et al., 2021). Moreover, noncoding regions (introns) are frequently transcribed to RNA within the cell before being spliced from the functional region before translation (Hawkins, 1996). Therefore, it might even be possible to use primers that target noncoding regions, such as the *trn*L (UAA) intron (Taberlet et al., 2007) in prey RNA studies, although the overall detection probability will likely be lower compared to coding regions.

The proposed prey RNA approach requires a measure of target fragment quantities to compare the relative DNA and RNA content within a sample. Here, we used RFU values produced by capillary electrophoresis (celPCR), which is a feasible and sensitive method for relative target fragment quantification (Thalinger et al., 2021). Alternatively, qPCR, also frequently used for eRNA quantification (Marshall et al., 2021; Wood et al., 2020), or droplet digital PCR (Wood et al., 2019) could be used. Moreover, the implementation of RNA in prey metabarcoding needs to be investigated.

Apart from methodological aspects that need to be considered in future studies with prey RNA, several biological factors might affect the detectability of prey RNA. First, the age of carrion will be an important factor that needs to be addressed. Previous studies have shown that prey DNA detectability decreases with carrion age (Foltan et al., 2005; Juen & Traugott, 2005). In the case of prey RNA, it will be especially interesting to determine the level of decay that makes carrion recognizable as such, that is, when RNA deterioration has progressed far enough to show a sufficiently high difference to prey DNA. This will likely also affect the observed RNA:DNA ratio. Further, both the predator (Hosseini et al., 2008; von Berg et al., 2008) and the prey identity (Eitzinger et al., 2014; Foltan et al., 2005; Wallinger et al., 2013) as well as meal size (Foltan et al., 2005; Hoogendoorn & Heimpel, 2001; Thalinger et al., 2017), or a mixed diet (Sint et al., 2018) can affect detection probability of prey DNA, factors that will possibly also impact RNA detections and the RNA:DNA ratios. Moreover, it is so far unknown how the mixing of carrion and fresh prey will affect the results of the presented approach.

In conclusion, we demonstrated that prey RNA can be detected in gut content samples after extended periods post‐feeding if fresh prey was consumed, but only very briefly and at strongly reduced amounts after carrion prey consumption. The analysis of prey RNA in parallel to prey DNA, therefore, holds great promise to differentiate between trophic links involving feeding on carrion and fresh prey. The prey RNA approach will apply to diagnostic and metabarcoding techniques alike and bears relevance for a wide range of different food web systems. While further research on this topic is anticipated, we suggest this novel approach will be of high value to assess the significance of scavenging for populations, communities, and ecosystems.

## AUTHOR CONTRIBUTIONS

Michael Traugott came up with the conceptual idea for the present research, Michael Traugott and Veronika Neidel led the funding acquisitions, Daniela Sint and Corinna Wallinger developed the methods, Veronika Neidel and Corinna Wallinger carried out the feeding experiments, Veronika Neidel carried out the molecular and statistical analysis and led the writing of the manuscript.

## CONFLICT OF INTEREST

The authors declare no conflict of interest.

## BENEFIT‐SHARING STATEMENT

Benefits from this research derive from sharing the data and results on a database as described above.

## Supporting information


Table S1‐S5

## Data Availability

Original data of the feeding experiment, including uncorrected RFU values, is available on Dryad (https://doi.org/10.5061/dryad.m37pvmd3d) (Neidel et al., 2022).
