# Supplementary material for: RNA allows identifying the consumption of carrion prey
Source: Mol Ecol Resour. 2022 Jun 20;22(7):2662–71. doi: 10.1111/1755-0998.13659 (PMC9541938; doi:10.1111/1755-0998.13659)
Supplement: Supplementary file 1 — Table S1‐S5 [file MEN-22-2662-s001.docx]

# Supporting Information

**RNA allows identifying the consumption of carrion prey**

Short running title: **RNA reveals carrion prey**

Veronika Neidel, Daniela Sint, Corinna Wallinger, Michael Traugott

*Applied Animal Ecology, Department of Zoology, University of Innsbruck, Technikerstraße 25, 6020 Innsbruck, Austria*

**Corresponding author:**

DI Veronika Neidel

E-Mail: veronika.neidel@uibk.ac.at

## Supplementary Information: Results

### Table S1: Number of regurgitates in feeding experiments with Harpalus rufipes that had fed on Drosophila melanogaster, either as fresh prey or 24-hour dead carrion. Regurgitation to collect gut content of beetles was elicited at five time points after feeding, each on different batches of carabids.

| ***Digestion time*** | |  | |  | |
| --- | --- | --- | --- | --- | --- |
|  |  | ***No. of H. rufipes fed with*** | | |  |
| *[h]* | | *fresh fly* | *carrion fly* | | *Total N* |
| ***0*** | | 10 | 10 | | 20 |
| ***6*** | | 10 | 13 | | 23 |
| ***12*** | | 10 | 12 | | 22 |
| ***24*** | | 13 | 13 | | 26 |
| ***48*** | | 11 | 14 | | 25 |
| *Total* | *54* | | *62* | | *116* |

### Table S2: Proportion of regurgitate samples from Harpalus rufipes testing positive for Drosophila-specific prey RNA or DNA after consumption of fresh or carrion fly.

|  |  | Fresh prey | Carrion prey |
| --- | --- | --- | --- |
|  | **digestion time** | **% of samples positive** | **% of samples positive** |
| **RNA** | 0 | 100 | 90 |
|  | 6 | 90 | 23.1 |
|  | 12 | 70 | 16.7 |
|  | 24 | 53.8 | 0 |
|  | 48 | 9.1 | 7.1 |
| **DNA** | 0 | 100 | 100 |
|  | 6 | 100 | 100 |
|  | 12 | 80 | 83.3 |
|  | 24 | 76.9 | 0 |
|  | 48 | 36.4 | 14.3 |

### Table S3: Pairwise comparison of the relative amount of Drosophila-specific prey DNA and RNA (signal strengths measured in Relative Fluorescent Units (RFU)) within regurgitates of Harpalus rufipes at different points in time after feeding on (a) fresh prey and (b) carrion prey via Wilcoxon Signed-Ranks tests, with r being the effect size, p the significance value (* < 0.05, ** < 0.01, *** < 0.001) and n, the sample number of the comparison. Test statistics omitted for groups with corrected RFU-values (24, 48 h).

|  | **Digestion time** | **DNA RFU mean ± sd** | **RNA RFU mean ± sd** | **r** | **p** | **n** | |
| --- | --- | --- | --- | --- | --- | --- | --- |
| 1. Fresh Prey | 0 | 2.61 ± 0.48 | 2.52 ± 0.7 | -0.02 | 0.919 | 20 |  |
|  | 6 | 1.74 ± 0.58 | 1.26 ± 1.11 | -0.29 | 0.193 | 20 |  |
|  | 12 | 1 ± 0.91 | 0.87 ± 0.94 | -0.16 | 0.496 | 18 |  |
|  | 24 | 0.6 ± 0.57 | 0.43 ± 0.97 | -0.27 | 0.197 | 12 |  |
|  | 48 | 0.08 ± 0.14 | 0.01 ± 0.04 | -0.54 | 0.125 | 8 |  |
|  |  |  |  |  |  |  |  |
| 1. Carrion Prey | 0 | 3.12 ±1.06 | 0.84 ± 1.05 | -0.65 | 0.004 ** | 20 |  |
|  | 6 | 1.19 ± 0.93 | 0.22 ± 0.62 | -0.72 | 0.000 *** | 26 |  |
|  | 12 | 0.56 ± 0.75 | 0.04 ± 0.11 | -0.63 | 0.005 ** | 20 |  |
|  | 24 | 0 ± 0 | 0 ± 0 | *NA* | *NA* | 0 |  |
|  | 48 | 0.04 ± 0.12 | 0.04 ± 0.14 | *NA* | *NA* | 6 |  |

### Table S4: Results of generalized linear models (GLM) for detection probability of Drosophila melanogaster consumption in regurgitates of Harpalus rufipes over digestion time. Individual models for different prey types, (A) fresh prey and (B) carrion prey, and target molecules, (1) prey DNA or (2) prey RNA, and over-all models for (C) each target molecule to reveal the effect of prey type (fresh, carrion) on detection probability of either molecular target.

|  |  | **Estimate** | **CI 95 %** | | **Std Error** | **z-value** | **p-value** |
| --- | --- | --- | --- | --- | --- | --- | --- |
|  |  |  | **2.5%** | **97.5%** |  |  |  |
| **A1. Fresh prey: DNA Detection probability** | | |  |  |  |  |  |
|  | Intercept | 3.252 | 1.907 | 5.052 | 0.785 | 4.141 | 0.000 |
|  | dig_time | -0.082 | -0.134 | -0.039 | 0.024 | -3.456 | 0.001 |
| **A2. Fresh prey: RNA Detection probability** | | |  |  |  |  |  |
|  | Intercept | 2.729 | 1.544 | 4.280 | 0.681 | 4.007 | 0.000 |
|  | dig_time | -0.109 | -0.178 | -0.060 | 0.029 | -3.740 | 0.000 |
| **B1. Carrion prey: DNA Detection probability** | | | |  |  |  |  |
|  | Intercept | 3.088 | 1.806 | 4.843 | 0.756 | 4.085 | 0.000 |
|  | dig_time | -0.157 | -0.250 | -0.091 | 0.040 | -3.885 | 0.000 |
| **B2. Carrion Prey: RNA Detection probability** | | | |  |  |  |  |
|  | Intercept | 0.362 | -0.576 | 1.371 | 0.490 | 0.739 | 0.460 |
|  | dig_time | -0.125 | -0.231 | -0.053 | 0.045 | -2.764 | 0.006 |
|  |  |  |  |  |  |  |  |
| **C1. DNA-Detection probability: DNA-binary ~ digestion_time + prey type** | | | | | | | |
|  | Intercept | 4.084 | 2.778 | 5.765 | 0.751 | 5.435 | 0.000 |
|  | digestion time | -0.110 | -0.154 | -0.075 | 0.020 | -5.502 | 0.000 |
|  | type carrion prey | -1.759 | -3.097 | -0.620 | 0.622 | -2.829 | 0.005 |
| **C2. RNA-Detection probability RNA-binary ~ digestion_time + prey type** | | | | | | | |
|  | Intercept | 2.825 | 1.741 | 4.168 | 0.611 | 4.621 | 0.000 |
|  | digestion time | -0.114 | -0.170 | -0.072 | 0.025 | -4.625 | 0.000 |
|  | type carrion prey | -2.547 | -3.778 | -1.486 | 0.579 | -4.403 | 0.000 |

### Table S5: Ratios of prey RNA- and prey DNA-RFU values produced for prey specific PCR products within the same sample, summarised per time interval after feeding on one fresh (fresh prey) or one carrion fruit fly, D. melanogaster (carrion prey) in regurgitates of H. rufipes. ‘W’ is the test statistic and ‘r’ the effect size of Wilcoxon-Ranked sum pairwise comparisons between the ratios of the different prey types.

|  | **ratio RFU_RNA_:RFU_DNA_** | | | | | | | |  |  |  |  |
| --- | --- | --- | --- | --- | --- | --- | --- | --- | --- | --- | --- | --- |
|  | ***fresh prey*** | | | | ***carrion prey*** | | | | **WRS test** | | |  |
| **dig. time**  **[h]** | ***mean*** | ± | ***sd*** | ***n*** | ***mean*** | ± | ***sd*** | ***n*** | ***W*** | ***r*** | ***p-value*** | |
| **0** | 0.97 | ± | 0.19 | 10 | 0.31 | ± | 0.38 | 10 | 89.00 | -0.66 | 0.003 | ** |
| **6** | 0.71 | ± | 0.60 | 10 | 0.12 | ± | 0.27 | 13 | 109.00 | -0.58 | 0.006 | ** |
| **12** | 0.85 | ± | 1.01 | 8 | 0.15 | ± | 0.32 | 10 | 54.00 | -0.29 | 0.213 |  |
| **24** | 0.80 | ± | 1.27 | 10 |  |  |  | 0 |  |  |  |  |
| **48** | 0.20 | ± | 0.41 | 4 | 0.00 | ± | 0.00 | 2 | 3.00 | -0.51 | 0.643 |  |
